# Medicine as a moral activity

**DOI:** 10.1080/02813432.2023.2285470

**Published:** 2024-02-07

**Authors:** Christopher Dowrick

**Affiliations:** University of Liverpool, Liverpool, UK

Are diseases things? And if so, what sort of things are they? Are they objective, indifferent entities, which doctors should seek to observe, understand and cure? Or are they phenomena, interactive entities, to be interpreted by the person (perhaps with the help of doctors) in the light of their experiences and biography, their life goals and values? Are diseases scientific, or moral?

These are the questions Eivind Meland and Stefan Hjörleifsson wrestle with in their fascinating discussion piece, published in this issue of the Scandinavian Journal of Primary Health Care and ably illustrated by a humorous Socratic dialogue between triathlon runner Thomas and his reflective general practitioner [1].

Meland and Hjörleifsson draw our attention to two principal philosophical sources.

French physician-philosopher Georges Canguilhem conceived organisms (including human beings) not on the basis of mechanical models but rather on the basis of their ability to adapt themselves to the challenges posed by their environment. He understood the art of medicine as a continual struggle between the descriptive (the act of producing evidence free of values) and normative or moral (the interpretation of evidence according to a set of values) [2,3]. And he saw life as a source of value, concerned with the full range and extent of human experiences, based on a sense of normative behaviour and social justice; of reflection and critical questioning, of engagement and (above all) of resistance.

Ian Hacking, Canadian philosopher of science, introduces us to the concept of ‘classificatory looping’. He explains how our methods of classifying people interact with the people being classified, and ultimately change the nature of these people. We are aware of being classified, in contrast to quarks, chemical elements or rock formations. We tend to ‘act under a description’: that is to say our ways of being ‘are by no means independent of the available descriptions under which [we] may act’ [4]. When we are aware of the classification we have been awarded, the way we experience ourselves changes. At the same time, those around us also react and behave differently to us as a consequence of the classification.

The debate between the GP and Thomas, who is seeking a cardiac rather than an existential explanation for his fatigue, is well understood along these lines. Is Thomas a ‘cardiac case’? Does he need urgent investigation for possible Wolff-Parkinson-White syndrome? Or may he benefit more from encouragement to reflect on his triathlon training programme, and the effects it may be having on his moral perspectives, on other valued elements of his life? What are the implications for him if his GP accedes to his request for a cardiology referral; or instead introduces him gently to the guiding principles of Acceptance and Commitment Therapy?

In conceptually similar vein, I continue to grapple with the decision whether or not to prescribe antidepressant medication, with the aim of enabling a person with symptoms of depression to return to their normal range of function. In so doing the risk is that they may lose their sense of ‘being normal’, and their sense of agency, precisely because they are having to rely on an external agent in the form of a daily or twice-daily tablet. They may wish to stop taking this medication when they feel better, but fear the consequences of so doing and hence decide to play safe and continue to take it. If antidepressants are not as useful as is commonly supposed, then such loss of personal agency and increase in fearfulness become important iatrogenic effects [5].

As GPs, we do well to adopt what Meland and Hjörleifsson describe as ‘respectful curiosity’. In the words of British psychoanalyst Wilfred Bion, we need to listen ‘without memory or desire’ [6]. When we listen with memory, we are intent on making the patient part of our old agenda; when we listen with desire we are intent on making them part of a new one. To listen purely, to just listen, is the most valuable thing; but also the most difficult. And then Ronald Epstein encourages us to turn toward suffering: actively seek to recognise it, become curious about the patient’s experience, and intentionally become more present and engaged [7].

We can extend these perspectives with the help of contemporary philosopher Havi Carel, who uses phenomenology to explore how her own illness (as distinct from disease) modifies her body, her values, and her world [8]. In this context, Thomas’ perception of his illness may be studied by himself (in conversation with his GP) as a lived experience: a process which cannot fail to include its existential, ethical, and social dimensions.

This brings us to the heart – and the art – of medical generalism, so elegantly portrayed in Joanne Reeve’s new book [9]. Basing her argument on the assumption of our innate ‘creative capacity’, she proposes that the fundamental task of generalist care is to consider the self who responds creatively to the experience of illness. Quality of care rests not on the application of a plethora of (well-meaning) clinical guidelines or the published outcomes of randomised controlled trials, but instead on what she calls ‘inductive foraging’: seeking a deep and contextualised understanding of who the patient is (rather than what disease they might have) and the challenges they face; drawing together multiple kinds of knowledge to get a better handle on their unique case, and then exploring what resources and support might help them meet the challenges they deem most important. Responses to these challenges are co-produced by patient and clinician, then tested and adapted during the course of their long-term clinical relationship.

So, Thomas and his GP can look forward to a lively series of conversations as they acknowledge and enable his creative capacity, transforming him from a failing runner into a flourishing human being.
